# Editorial: Psychrotolerant microbes: ecological adaptations and biotechnological potential

**DOI:** 10.3389/fmicb.2026.1830631

**Published:** 2026-04-14

**Authors:** Anita Pandey, Avinash Sharma, Sergio Leiva Poveda

**Affiliations:** 1 Department of Biotechnology, Graphic Era (Deemed to be University), Dehradun, Uttarakhand, India; 2 BRIC-National Centre for Cell Science, Pune, Maharashtra, India; 3 Institute of Biochemistry and Microbiology, Faculty of Sciences, Universidad Austral de Chile, Valdivia, Chile

**Keywords:** adaptations, Antarctic, Arctic, biotechnological significance, Himalaya, psychrophiles

Microbial colonization of extreme environments, particularly by bacteria and archaea, has gained increasing attention from the scientific community worldwide as it advances understanding of their diversity, adaptive mechanisms, and potential applications ranging from ecological to biotechnological processes (Pandey and Sharma, 2021; Zannoni et al., 2022). Extreme environments encompass a wide range of habitats defined by one or more physicochemical and ecological constraints that limit biological survival, reproduction, and activity.

The Earth is dominated by a large proportion of low-temperature environments, both terrestrial and aquatic. Polar and high mountain regions make up a substantial share of Earth's land surface, and vast areas of the planet are covered by glaciers, permafrost, snow, and ice. These areas, collectively termed the cryosphere, are widely recognized as biodiversity hotspots of global ecological importance (Intergovernmental Panel on Climate Change, 2022). Bioprospecting of these distinct cold ecosystems has revealed a plethora of microorganisms (psychrophiles and psychrotolerants), many of them novel and often endemic, exhibiting fascinating adaptive strategies. Cold-adapted microorganisms undergo several modifications at cellular and biochemical levels to alleviate the influence of low-temperature stress conditions (Hualpa-Cutipa et al., 2022; Chauhan et al., 2023).

This Research Topic “*Psychrotolerant microbes: ecological adaptations and biotechnological potential*,” aims to summarize the functional versatility of cold-adapted microorganisms, with particular reference to their peculiarities, along with the ecological and biotechnological significance, through selected articles. This has enriched our knowledge on the structural and functional aspects of cold-adapted microorganisms, originally isolated from areas including the Arctic, the Antarctic, the Himalaya, ancient cave ice and the Greenland Ice Sheet.

Díez et al. addressed the topic “*Environmental distribution, physiology, and genomic adaptations of arctic ammonia-oxidizing archaea (AOA)*,” referring to them as the main drivers of nitrification in Arctic soils under the influence of warming temperatures—a consequence of climate change. Compared to other terrestrial environments, the article

reports the low diversity of AOA in Arctic soils, consisting of only two specific clades detected in most Arctic ecosystems. An isolate of *Nitrosocosmicus arcticus* has been demonstrated to actively oxidize ammonia at 4 °C—the lowest reported temperature so far. Rappazzo et al. examined Arctic and Antarctic lake sediments and reported contrasting contaminant profiles and prokaryotic assemblages, highlighting the role of persistent organic pollutants (POPs) and trace metals as key drivers of ecological change in polar environments.

Castilho et al. reported the isolation of *Psychrobacillus antarcticus* Val9 from Antarctic soil with a bioemulsifier showing anticorrosion potential, and addressed the challenging industrial problem of corrosion, particularly in oilfield infrastructure. This study highlighted the anticorrosive, antibiofilm, and biocidal properties of two bioproducts: a bioemulsifier from *P. antarcticus* Val9 and a surfactin from *Bacillus velezensis* H2O-1, used to simulate water injection header conditions. Further, Zhang et al. employed high-throughput sequencing to characterize bacterial community composition and function across habitats in the Antarctic Fildes region, within the broader context of global climate change.

Reporting the genome sequence and functional profile of *Psychrobacter* strain SC65A.3, preserved in 5,000-year-old cave ice, Paun et al. provided insights into its ancient resistome, antimicrobial potential, and enzymatic activities. Jaarsma et al. reported the encoded and expressed biosynthetic potential of Greenland Ice Sheet microbes, highlighting their potential in producing natural products with ecological and biotechnological applications.

Tamang et al. studied the bacterial diversity in the Kabru Glacier ice cores of Sikkim Himalaya under the influence of multiple environmental parameters. This study further suggests the need to investigate parameters such as the occurrence of antibiotic-resistant and metal-tolerant bacteria to develop a better understanding on microbial adaptation and potential ecological implications in high-altitude glacial ecosystems.

The contributions to this Research Topic emphasize the relevance of psychrophilic microorganisms adapted to diverse ecological niches, covering themes that span ecology and biotechnology. Collectively, the articles indicate that bioprospecting in the cryosphere is expanding through varied approaches and methodologies. The capacity of these microbes to produce enzymes and bioactive compounds reflects adaptive responses that underpin their potential applications in agriculture, medicine, and industrial processes. In this context, the conservation of cold ecosystems and their native microbial communities is crucial, not only for preserving their ecological functions and resilience to environmental change, but also for safeguarding the genetic and functional richness that underlies this biotechnological potential. Continued exploration and bioprospecting efforts targeting microbial diversity in poorly explored cold environments will remain important for advancing such endeavors (Chauhan and Pandey, 2024).
